# Differences in outpatient care and treatment utilization for patients with HIV/HCV coinfection, HIV, and HCV monoinfection, a cross-sectional study

**DOI:** 10.1186/1471-2334-14-217

**Published:** 2014-04-23

**Authors:** Terence L Johnson, Joshua C Toliver, Lu Mao, Christine U Oramasionwu

**Affiliations:** 1 University of North Carolina, UNC Eshelman School of Pharmacy, Division of Pharmaceutical Outcomes and Policy, Chapel Hill, NC 27599-7355, 2215 Kerr Hall, USA; 2 University of North Carolina, UNC Gillings School of Global Public Health and the UNC Center for AIDS Research (CFAR) Biostatistics Core, Chapel Hill, NC, USA

**Keywords:** HIV, HCV, Coinfection, Antiretroviral, Antiviral, Therapy, Utilization

## Abstract

**Background:**

Few studies have explored how utilization of outpatient services differ for HIV/HCV coinfected patients compared to HIV or HCV monoinfected patients. The objectives of this study were to (1) compare annual outpatient clinic visit rates between coinfected and monoinfected patients, (2) to compare utilization of HIV and HCV therapies between coinfected and monoinfected patients, and (3) to identify factors associated with therapy utilization.

**Methods:**

Data were from the 2005–2010 U.S. National Hospital Ambulatory Medical Care Surveys. Clinic visits with a primary or secondary ICD-9-CM codes for HIV or HCV were included. Coinfection included visits with codes for both HIV and HCV. Monoinfection only included codes for HIV or HCV, exclusively. Patients <15 years of age at time of visit were excluded. Predictors of HIV and HCV therapy were determined by logistic regressions. Visits were computed using survey weights.

**Results:**

3,021 visits (11,352,000 weighted visits) met study criteria for patients with HIV/HCV (8%), HIV (70%), or HCV (22%). The HCV subgroup was older in age and had the highest proportion of females and whites as compared to the HIV/HCV and HIV subgroups. Comorbidities varied significantly across the three subgroups (HIV/HCV, HIV, HCV): current tobacco use (40%, 27%, 30%), depression (32%, 23%, 24%), diabetes (9%, 10%, 17%), and chronic renal failure (<1%, 3%, 5%), (*p* < 0.001 for all variables). Annual visit rates were highest in those with HIV, followed by HIV/HCV, but consistently lower in those with HCV. HIV therapy utilization increased for both HIV/HCV and HIV subgroups. HCV therapy utilization remained low for both HIV/HCV and HCV subgroups for all years. Coinfection was an independent predictor of HIV therapy, but not of HCV therapy.

**Conclusion:**

There is a critical need for system-level interventions that reduce barriers to outpatient care and improve uptake of HCV therapy for patients with HIV/HCV coinfection.

## Background

Human Immunodeficiency Virus (HIV) and hepatitis C virus (HCV) monoinfection have been the subjects of ample research over the past two decades; however, HIV/HCV coinfection has only recently been documented as a growing medical concern in the United States [1]. Combination HIV antiretroviral therapy and combination HCV antiviral therapy have been recommended since the late 1990s, as they each greatly reduce patient morbidity and mortality [2,3]. While antiretroviral and antiviral therapies are widely recommended for use in patients with coinfection [4], these patients continue to experience poorer health outcomes than those with monoinfection. For instance, these individuals are at increased risk for accelerated progression of liver disease and increased rates of morbidity and mortality [5,6].

If patients with coinfection do not utilize outpatient services to the extent that patients with HIV or HCV utilize these services, consequently, patients with coinfection may not be prescribed therapy to the extent that patients with monoinfection are prescribed therapy. Few studies to date have explored how outpatient health care utilization patterns differ by infection status. Thus, the extent to which patients with coinfection receive care in the U.S. outpatient health care delivery system, as compared to patients with monoinfection, is relatively unknown. The U.S. health care system is based on a multi-payer system, whereby, medical care is provided by various independent organizations, rather than a single universal entity. These independent organizations are largely owned and operated by the private sector; however, other players in the market include the U.S. government and other non-profit organizations [7]. The multitude of health care providers can result in various barriers to care, including financial barriers for individuals who are uninsured or underinsured, lack of availability of specialized professionals, and inability to reach providers [7].

The objectives of this study were to compare, between patients with HIV/HCV coinfection and monoinfection (HIV and HCV), (1) annual outpatient clinic visit rates, (2) trends in yearly outpatient prescription for HCV antiviral therapy and HIV antiretroviral, (3) conduct multivariate analysis to identify factors associated with antiretroviral and antiviral utilization.

## Methods

### Data Source

The 2005–2010 U.S. National Hospital Ambulatory Medical Care Surveys (NHAMCS) were used for this study. The NHAMCS are nationwide probability sample surveys that are conducted annually by the Centers for Disease Control and Prevention (CDC). The NHAMCS are representative of approximately 500 general and pediatric hospital outpatient clinics, while excluding federal, military, veteran affairs, and institutional hospital clinics. Survey data are available to the public and are a national representation of annual clinic visit records. The objective of the NHAMCS is to provide a population-level estimate of the utilization of outpatient services in the United States, therefore, some patient level data, such as laboratory results, are not always available. Each record consists of up to three International Classification of Diseases, Ninth Revision, Clinical Modification (ICD-9-CM) diagnosis codes and medication codes that document up to eight medications listed at time of the clinic visit. The outpatient clinic visits are systematically sampled using a multi-stage process conducted by trained field representatives (e.g., health care provider or clinic staff) during a randomly assigned 4-week period. The various study survey error rates typically range between 0.3% and 0.9%; however, independently selected quality control samples, approximately 10% of patient record forms, are keyed and coded. Additional information about the NHAMCS data collection process and interpretation are available elsewhere [8].

### Study design

This was a nationally representative, retrospective, cross-sectional study. All variables were retrieved from the NHAMCS and included patient demographics (patient age at time of clinic visit, gender, race/ethnicity, geographic region in the United States, insurance status) and visit characteristics (established patient, patient’s primary physician/provider, visit diagnosis, year of visit, and providers seen). Insurance status was classified as private, Medicare, Medicaid, self-pay, no charge, or unknown/other. In the United States, the major insurance payers are private insurance and government in the form of Medicare and Medicaid [9,10]. Although both Medicare and Medicaid are examples of government-funded programs, they are funded differently. Medicare is a national program designed to provide medical care for older adults and is funded by the U.S. government. Patients aged 65 or older, with end-stage renal disease, or with certain qualifying disabilities are eligible for Medicare. In contrast, Medicaid is a state-specific program designed to provide health care to low-income patients and is funded on the federal and state level. Patients are eligible for this program if they meet certain criteria, however, this criteria can vary greatly based on the individual state.

Comorbidities were based on additional clinic visit diagnoses to include: chronic renal failure, depression, diabetes, and current tobacco use. All clinic visits with a primary or secondary ICD-9-CM diagnosis code for HIV or HCV were included. The following ICD-9-CM codes were used to identify HIV infection: 042, V08, and 079.53 and the following codes were used to identify HCV infection: 070.41, 070.44, 070.51, 070.54, 070.70, and 070.71. HIV monoinfection visits excluded HCV ICD-9-CM codes and HCV monoinfection visits excluded HIV ICD-9-CM codes. HIV/HCV coinfection visits included those that had ICD-9-CM codes for both HIV and HCV.

Medication drug codes were determined from the National Center for Health Statistics (NCHS) drug database to define HCV antiviral medications and HIV antiretroviral medications. HCV antiviral medications included any of the following: ribavirin, interferon, or pegylated interferon. HIV antiretroviral medications included any of the following: nucleoside reverse transcriptase inhibitors, non-nucleoside reverse transcriptase inhibitors, protease inhibitors, integrase inhibitors, entry inhibitors, CCR5 antagonists, or other antiretroviral combination products.

Patients under the age of 15 at time of the clinic visit were excluded. The Ethics Review Board of NCHS approves the NHAMCS on an annual basis. The University of North Carolina Office of Human Research Ethics determined that this project was not considered Human Subjects Research according to regulatory criteria, and, therefore, institutional review board approval was not needed.

### Data analysis

Survey weights for each observation in the survey sample were used to generate national estimates for clinic visits. The survey weights are calculated by NCHS and are the result of corresponding sampling fractions at each stage in the sample design. The study weights are adjusted for nonresponse within time of year, geographical region, and urban/rural and ownership designations, producing an unbiased national estimate of outpatient visit occurrences, percentages, and characteristics. In addition, weighted estimates account for the cluster and stratum effect of the primary sampling unit (PSU). Weighted clinic visit estimates and disease surveillance estimates were used to calculate annual clinic visit rates (visits per U.S. population with diagnosed infection).

All data analyses were conducted using JMP® 8.0 and SAS® 9.2 (SAS Institute Inc., Cary, NC). A two-tailed alpha-level <0.05 was used to determine statistical significance. Demographic characteristics and select comorbidities were compared across disease groups (HIV/HCV, HIV, HCV). Survey logistic regressions were performed to determine factors associated with utilization of HIV therapy and utilization of HCV therapy. Coinfection status was entered into both models as an indicator variable, while adjusting for relevant demographic covariates. SAS procedures SURVEYFREQ, SURVEYMEANS, and SURVEYLOGISTIC were used where appropriate.

## Results

Approximately, 11,352,000 clinic visits (3,021 unweighted observations) met study criteria for patients with HIV/HCV coinfection (8%), HIV monoinfection (70%), or HCV monoinfection (22%) between 2005 and 2010. A comparison of demographics, select comorbidities, and outpatient visit characteristics by infection group is presented in Table 1. In general, the HCV group was older in age and had the highest proportion of females and whites as compared to the HIV/HCV and HIV groups. Clinic visits predominantly occurred in the southern United States and the most common form of insurance coverage listed across all groups was Medicaid, followed by Medicare. The following comorbidities varied significantly across the three groups (HIV/HCV, HIV, HCV): current tobacco use (40%, 27%, 30%), depression (32%, 23%, 24%), diabetes (9%, 10%, 17%), and chronic renal failure (<1%, 3%, 5%), (*p* < 0.001 for all variables). The majority of patients had previously established care at the clinic that they visited. Physicians often provided care at these visits (approximately 80% for all three groups). By comparison, nurse practitioners and physician assistants provided care less often. In contrast, nurses provided care at approximately two-thirds of visits for the HIV/HCV and HIV groups, but only provided care at half of visits for the HCV group (*p* < 0.001). It was more common for those with HIV/HCV to visit with their primary care provider/physician, as compared to those with either HIV monoinfection or HCV monoinfection.

**Table 1:** Comparison of demographics, select comorbidities, and outpatient visit characteristics in patients with HIV/HCV, HIV, and HCV

**Characteristic**	**HIV/HCV coinfection**	**HIV monoinfection**	**HCV monoinfection**	***P*-value**
No. of Unweighted Observations	200	1992	829	
No. of Visits in 1000s (95% CI)	859 (422–1296)	7,926 (4,772–11081)	2,567 (1658–3476)	***<0.001***
Proportion of Study Visits	8%	70%	22%	
***Patient demographics***	-	-	-	
Age (years), mean (95% CI)	46.4 (43.1–49.7)	42.9 (41.6–44.2)	50.4 (49.0–51.9)	***<0.001***
Gender	-	-	-	***<0.001***
Male (%)	64	68	59	-
Female (%)	36	32	41	***-***
Race/ethnicity (%)	-	-	-	***<0.001***
White	31	25	56	-
African-American	49	50	29	-
Hispanic	18	23	11	-
Other	1	2	4	-
Geographic region (%)	-	-	-	***<0.001***
Northeast	30	26	28	-
Midwest	11	18	9	-
West	7	12	10	-
South	52	43	53	-
Insurance status (%)	-	-	-	0.051
Private	4	16	23	-
Medicare	20	16	14	-
Medicaid	45	43	31	-
Other/unknown	11	11	10	-
Self-pay	3	4	8	-
No Charge	17	9	14	-
***Comorbidities***	-	-	-	-
Chronic Renal Failure (%)	<1	3	5	***<0.001***
Depression (%)	32	23	24	***<0.001***
Diabetes (%)	9	10	17	***<0.001***
Current Tobacco Use (%)	-	-	-	***<0.001***
Yes	40	27	30	-
No	28	28	38	-
Unknown	32	45	32	-
***Visit characteristics***	-	-	-	-
Established Patient (%)	94	93	83	***<0.001***
Patient’s Primary Care Physician/Provider (%)	-	-	-	***<0.001***
Yes	48	36	35	-
No	49	58	60	-
Unknown	3	6	5	-
Providers Seen (%)	-	-	-	-
Physician	81	79	80	0.5
RN/LPN	63	64	54	***<0.001***
Nurse practitioner/midwife	14	13	14	0.2
Physician assistant	4	4	4	0.6
Other	23	25	20	***<0.001***

**Figure 1:**
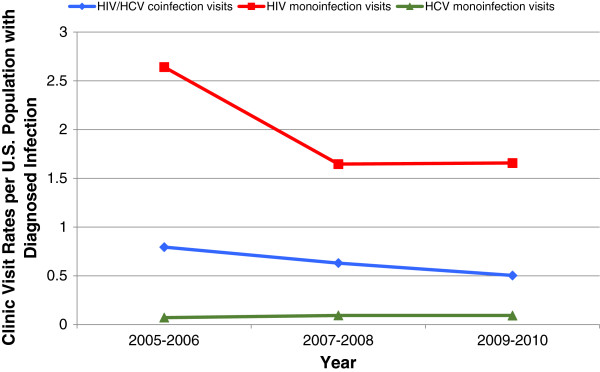
Trends in annual outpatient clinic visit rates for patients with HIV/HCV, HIV, or HCV infection.

**Figure 2:**
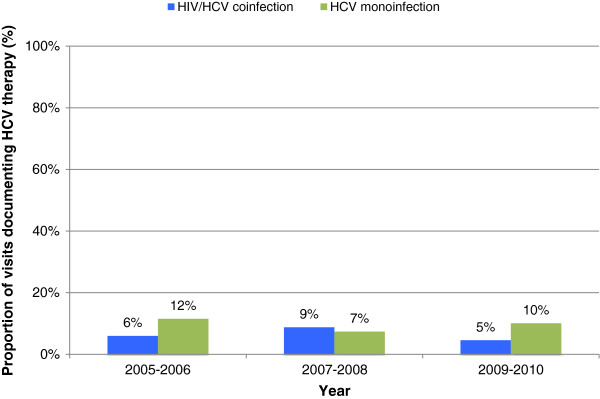
Clinic visits that documented HCV antiviral therapy prescription (HIV/HCV vs. HCV).

**Figure 3:**
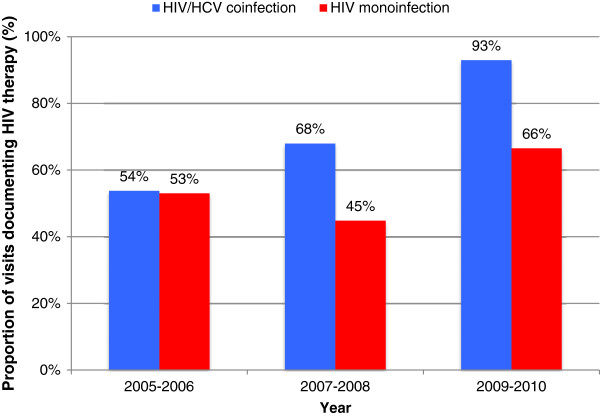
Clinic visits that documented HIV antiretroviral therapy prescription (HIV/HCV vs. HIV).

For all study years, annual visit rates were highest in those with HIV, followed by HIV/HCV (Figure 1). Annual visit rates were consistently lower in those with HCV. Documentation of HCV antiviral therapy utilization remained low for both HIV/HCV and HCV groups for all years (Figure 2). Results from the logistic regression to identify predictors of HCV therapy are presented in Table 2. Presence of depression as a comorbidity was associated with decreased odds of HCV therapy (adjusted odds ratio [95% CI]; 0.265 [0.131 - 0.536]). HIV/HCV coinfection (compared to HCV monoinfection) was not associated with HCV therapy. HIV antiretroviral therapy utilization increased for both HIV/HCV and HIV groups; the increase was more pronounced in the HIV/HCV group (Figure 3). Per regression analysis in Table 3, HIV/HCV coinfection (compared to having HIV alone) was associated with increased odds of HIV therapy (*p* < 0.01 in all years). Negative predictors of HIV therapy included African-American race/ethnicity (*p* = 0.045) and no charge for the clinic visit (*p* = 0.044).

**Table 2:** Multivariate regression analysis of factors associated with HCV antiviral therapy

	**Adjusted OR (95% CI)**	***P*-value**
**Age**	0.969 (0.934, 1.005)	0.0892
**Gender**		
Female	1	-
Male	0.805 (0.346, 1.872)	0.6138
**Race**		
White, non Hispanic	1	-
African-American, non Hispanic	1.019 (0.399, 2.603)	0.3692
Hispanic	1.882 (0.671, 5.28)	0.3924
Other	1.93 (0.515, 7.237)	0.4715
**Region**		
Northeast	1	-
Midwest	1.734 (0.368, 8.172)	0.6205
West	0.920 (0.269, 3.144)	0.438
South	1.857 (0.686, 5.026)	0.277
**Insurance**		
Private Insurance	1	-
Medicare	3.373 (0.665, 17.097)	0.1527
Medicaid	2.257 (0.481, 10.592)	0.5098
Other/unknown	1.300 (0.204, 8.276)	0.4562
Self-pay	2.440 (0.559, 10.644)	0.4657
No Charge	1.903 (0.494, 7.335)	0.9823
**Depression**	0.265 (0.131, 0.536)	***0.0002***
**HIV/HCV Coinfection vs. HCV Monoinfection**		
2005-2006 (year of visit)	0.5145 (0.148, 1.789)	0.296
2007-2008 (year of visit)	0.3082 (0.014, 6.943)	0.4589
2009-2010 (year of visit)	0.3293 (0.039, 2.786)	0.3079

**Table 3:** Multivariate regression analysis of factors associated with HIV antiretroviral therapy

	**Adjusted OR (95% CI)**	***P*-value**
**Age**	1.012 (0.998, 1.025)	0.1024
**Gender**		
Female	1	-
Male	1.306 (0.823, 2.073)	0.2571
**Race**		
White, non Hispanic	1	-
African-American, non Hispanic	0.682 (0.500, 0.932)	0.4559
Hispanic	0.795 (0.486, 1.301)	0.9695
Other	0.759 (0.211, 2.725)	0.9097
**Region**		
Northeast	1	-
Midwest	1.271 (0.545, 2.966)	0.211
West	0.442 (0.137, 1.421)	0.098
South	1.148 (0.589, 2.238)	0.2706
**Insurance**		
Private Insurance	1	-
Medicare	0.87 (0.588, 1.286)	0.1827
Medicaid	0.547 (0.298, 1.005)	0.0526
Unknown/other	0.602 (0.324, 1.119)	0.0531
Self-pay	0.521 (0.216, 1.258)	0.1352
No Charge	0.629 (0.398, 0.996)	***0.0437***
**HIV/HCV Coinfection vs. HIV Monoinfection**		
2005-2006 (year of visit)	2.533 (1.618, 3.965)	***<.0001***
2007-2008 (year of visit)	7.464 (1.9, 29.317)	***0.004***
2009-2010 (year of visit)	2.888 (1.364, 6.117)	***0.0056***

## Discussion

This is one of the first studies to compare outpatient utilization patterns for HIV/HCV coinfection, HIV monoinfection and HCV monoinfection on a national perspective. One advantage to the observational nature of the study design is the generalizability of the findings. The study evaluated patterns of care in the outpatient setting and are therefore, more reflective of actual clinical practice as compared to results from studies that are conducted in a controlled setting. This study reveals that there are differences in utilization of outpatient services based on infection status (coinfection vs. monoinfection). Differences in demographic characteristics across the three groups noted in this study are consistent with prior studies. Patients with HIV tended to be younger in age [11], of male gender [11,12], and of African-American race [11,12], as compared to patients with HCV.

An unexpected finding was the variation of clinic visit rates by infection type; there were more visits for patients with HIV than for patients with HCV, including those with coinfection. National surveillance data estimate that approximately 5.2 million persons in the United States are chronically infected with HCV, whereas approximately 1.1 million persons in the United States are infected with HIV [13,14]. The epidemiologic burden of HCV is greater than that of HIV, but utilization of outpatient care was relatively low for this patient population. In addition, it is estimated that one quarter of individuals living with HIV are coinfected with HCV [15]. Given that HIV requires lifelong management, and therefore necessitates routine outpatient care, one would expect that patients with HIV/HCV visit the clinic at least to the same extent as patients with only HIV. From a public health standpoint, this implies that coinfected individuals are not entering into care at the same rate as HIV monoinfected individuals. Low utilization of outpatient services precludes coinfected patients from receiving timely antiviral or antiretroviral therapies, leading to increased utilization of acute care services such as emergency department visits and hospital admissions [16]. These findings highlight the need to reduce entry to care barriers for this patient population.

Outpatient prescriptions for antiviral therapy was quite low across both groups with HCV. However, utilization was slightly lower for those with coinfection compared to those with monoinfection (7% vs. 10%, respectively). Uptake of HCV therapy has historically been low. Two separate studies by Cheung *et al.*[17] and Tsui *et al.*[18] sought to evaluate antiviral treatment rates for outpatient visits in patients with HCV, using national survey data. Both investigators reported that fewer than 10% of all HCV-related patient visits documented antiviral therapy. Data from these two studies were only through 2006, whereas data from the present investigation are through 2010. Nevertheless, the findings still persist; antiviral treatment rates in the United States have remained unchanged over the past several years. More efforts are needed to improve HCV treatment utilization rates.

The studies by Cheung *et al.* and Tsui *et al.* did not make a distinction for patients with monoinfection vs. patients with coinfection. Patients that are dually infected tend to experience accelerated progression of end-stage liver disease leading to increased risk of morbidity and mortality [5,6,19-21]. This distinction is important given their unique, clinical needs. Butt *et al.* conducted an investigation to compare treatment rates in patients with monoinfection vs. patients with coinfection [12]. Eligible patients were recruited and referred for HCV care to infectious diseases/HIV and hepatology clinics. Given that these patients were prospectively referred for HCV care to specialty clinics, it is not surprising that the overall treatment rate was relatively high at 50%. Nevertheless, the investigators determined that HCV treatment rates were lower in patients with coinfection compared to patients with monoinfection (32% vs. 62%; *p* < 0.001). Coinfection status was also independently associated with a decreased likelihood of HCV treatment, despite controlling for factors in multivariate analysis (adjusted OR 0.33, 95% CI 0.21-0.53). Lower treatment rates for patients with HIV/HCV receiving care in specialized settings have been reported elsewhere. Scott *et al.* conducted a retrospective evaluation of HCV therapy in a cohort of HIV patients receiving primary care at a HIV specialty clinic [22]. Only 16% of clinic patients ever received antiviral therapy. Similar proportions were noted in longitudinal data from the HIV Outpatient Study (HOPS); only 20% of 507 patients with confirmed coinfection initiated HCV treatment during the period of observation [6]. While an increasing proportion of HOPS participants were treated over the 3-year baseline periods in 1999–2001 (19%), 2002–2004 (21%) and 2005–2007 (28%), this overall rise was not statistically significant (*p* = 0.3). However, time for treatment initiation following confirmed HCV diagnosis decreased significantly (*p* < 0.001). Collectively, these findings underscore the ongoing issue of low HCV treatment uptake, particularly in patients with coinfection.

According to guideline recommendations, the primary goal of HCV therapy is to achieve a sustained virologic response (SVR) six months post treatment [23,24]. Such a response is effectively consistent with HCV cure for patients and greatly reduces progression to end-stage liver disease, hepatocellular carcinoma (HCC), and death [25]–[27]. To achieve this goal, therapy should be considered for all patients with chronic HCV. Pre-treatment assessments should be performed as certain conditions may preclude patients from receiving therapy. For instance, treatment is not recommended for patients with decompensated liver disease, severe uncontrolled psychiatric illness, and current alcohol and/or substance abuse [23]. Historically, antiviral treatment rates have been low due to concerns of side effects and/or adverse events associated with HCV standard of care with pegylated interferon and ribavirin (pegIFN + RBV). Reported adverse events often include hemolytic anemia associated with ribavirin use and influenza-like symptoms and neuropsychiatric effects associated with interferon use [23]. Fortunately, improved direct-acting antiviral (DAA) agents are associated with improved SVR rates and are now guideline-endorsed for use in combination with pegIFN + RBV [24]. The possibility of IFN-free regimens is also on the horizon [28]. For current clinical practice, particularly for those with coinfection, pegIFN + RBV are still a key component of the regimen. It has been projected that the addition of DAA agents to pegIFN + RBV will actually increase regimen complexity over the next few years as data emerge from clinical trials [20,29]. Other treatment considerations still include the concern for increased pill burden and the potential for drug-drug interactions with HCV medications and concomitant administration of antiretroviral therapy. Efforts are needed to improve HCV treatment utilization rates, particular in patients with coinfection.

Outpatient antiretroviral therapy increased in both groups with HIV. The goals of antiretroviral therapy include viral suppression, transmission prevention, and the restoration and preservation of immune function [30]. Since the eradication of the virus is not achieved by available antiretroviral medications, duration of HIV therapy is considered to be a lifelong commitment. It is expected that HIV therapy utilization was greater than HCV therapy utilization at the time of clinic visit. Other studies have reported high and/or rising use of HIV therapy, in both coinfection and monoinfection [6,11,31]. With regards to those with HIV/HCV, the majority (87%) of patients in the aforementioned HOPS study had some form of prior exposure to antiretroviral therapy [6]. High use is consistent with national treatment guidelines which now recommend antiretroviral therapy for all HIV-infected individuals, regardless of CD4 cell count, to reduce the risk of disease progression and for the prevention of the HIV transmission [30]. Fortunately, the advent of newer antiretroviral medications has lessened the concern for regimen complexity, drug-drug interactions, and pill burden. In the present study, the rise in antiretroviral coverage was more pronounced for those with coinfection, with the proportion rising from 54% in 2005–2006 to 93% in 2009–2010. This finding implies that HIV treatment is improving for this patient population. However, this only applies to patients that are in care. As described earlier, it was expected that the coinfection clinic visit rate would mirror that of the HIV monoinfection rate, but in fact, the rate was lower. Continued efforts are needed to engage patients diagnosed with HIV/HCV coinfection into outpatient care.

One of our findings was that African-American race/ethnicity was associated with lower odds of HIV antiretroviral therapy. Racial disparities in access to regular HIV care still persist; African-Americans have been shown to have sub-optimal engagement in the HIV continuum of care, particularly African-American males and African-American youth [32]. Gaston *et al.* recently conducted a systematic review to understand factors that influence engagement and adherence to HIV medical care among African-Americans [33]. A review of the 16 studies revealed that lack of social support, perceived discrimination and racism, and conspiracy beliefs about HIV and related treatments were barriers to HIV care, whereas, good quality relationships with health care providers facilitated adherence to HIV-related care [33].

Engagement in outpatient care is key for the management of both HIV and HCV. Despite recent and emerging advances in treatment, barriers to care persist, particularly for HCV care. The most common barriers are at the systems level (e.g., limited infrastructure for assessment and treatment, accessing care, high treatment costs), provider level (e.g., perceptions of poor patient adherence, concerns for active drug abusers, lack of experience treating patients), and at the patient level (e.g., lack of knowledge, misconceptions, level of motivation) [20,34]. Potential strategies to improve engagement in care include routine HCV testing and linking patients to care immediately following diagnosis. Furthermore, HCV care services can be expanded to other primary care services, which can be accomplished through cross-specialty provider education and training and patient pretreatment education [35]. Future research should delve further into outpatient utilization patterns to evaluate differences in contextual factors, adherence to prescribed medications, and patient-perceived barriers to care. A comprehensive approach that addresses these barriers can help to improve entry to outpatient care.

This study is subject to some limitations. The multivariate analysis conducted within this study should be interrupted carefully. The NHAMCS are designed to provide population-level estimates. Certain patient-levels factors that can be helpful in determining treatment initiation were unavailable. As such, multivariate analyses in this study did not adjust for HCV genotype, viral load, CD4 cell count, and patients’ medical history. Additionally, despite spanning 13 years, the study was not longitudinal and could not assess which patients were continuing with care over time. Findings represent only a snapshot in time and it is difficult to infer future trends. The NHAMCS data are presented as visit-level data rather than patient-level data; it is possible that the analysis captures patients that are sampled multiple times. However, only a patient returning to the clinic within the four-week reporting interval would potentially be sampled more than once. The NHAMCS survey instrument has a restriction on the maximum number of medications; no more than eight medications can be documented at time of visit. It is not uncommon for patients with HIV or HCV to use multiple medications. Therefore, we did not assess regimen appropriateness; rather, we evaluated documentation of any antiviral or antiretroviral medication. Up until 2005, medications were coded based on a 5-digit drug code. Starting in 2006, medications were coded based on a 6-digit code (in addition to the 5 digit code). We cross-referenced all codes to ensure accuracy in identifying medications.

Data regarding physician specialty (e.g., hepatologist, infectious diseases specialist) and detailed patient histories (e.g., past medication histories, prior treatment response, objective laboratory data) were not available in these surveys. Rather, we were able to assess patients that were visiting their primary care physician/provider as well as other types of health care professionals that provided care during the visit. Other providers in the U.S., such as nurse practitioners and physician assistants, have prescriptive authority when practicing under the direction of a supervising physician. Including the various types of providers seen is an indicator for a multidisciplinary approach to care for these patients. Moreover, we found that the majority of all patients had previously established care at the clinics that they visited, which is indicative of continued engagement in care at that facility.

## Conclusions

These data provide national, population-level estimates for health care utilization trends in patients with HIV/HCV, HIV, or HCV. Patients with coinfection have different medical needs compared to those with HIV or HCV alone. Nevertheless, prescription of HCV therapy remains low in the outpatient setting. While HIV therapy utilization was greater in patients with HIV/HCV than those with HIV, consistently lower clinic visit rates for the latter group reveal a disparity in the use of outpatient services. There is a critical need for system-level interventions that reduce barriers to care. Such efforts can improve treatment-related outcomes for this patient population. Future studies should investigate how health care utilization and HCV-related outcomes differ for patients with HIV/HCV compared to patients with HCV monoinfection, specifically in the context of DAA-based antiviral therapy.

## Competing interests

The authors declare that they have no competing interests.

## Authors’ contributions

CO was the senior investigator on the manuscript; she contributed substantially to the design of the research study, data acquisition, interpretation of data analysis, and drafting the manuscript. TJ contributed significantly to the study design, data analysis, data interpretation, organizing and drafting the manuscript. LM conducted the data analysis, assisted in interpretation of the analysis, and drafting the manuscript. JT participated in the study design, data acquisition, data analysis, and drafting the manuscript. All authors have reviewed and approved the final manuscript.

## Pre-publication history

The pre-publication history for this paper can be accessed here:

http://www.biomedcentral.com/1471-2334/14/217/prepub
